# Unraveling the Role of Microbes in Remediation of High Molecular Weight Polycyclic Aromatic Hydrocarbon Persistence in the Environment

**DOI:** 10.1007/s12010-025-05245-w

**Published:** 2025-05-03

**Authors:** Tony Hadibarata, Kitara Pranareswari Hadibarata

**Affiliations:** 1 Environmental Engineering Program, Curtin University, CDT250, 98009 Miri, Malaysia; 2 Knewton Global School, Jalan Desa Senadin, KBLD, 98000 MIRI, Malaysia

**Keywords:** High molecular weight polycyclic aromatic hydrocarbons, Microbial degradation, Bacteria, Microbial consortium, Anaerobic biodegradation, Biosurfactant

## Abstract

The persistence and recalcitrance of high molecular weight polycyclic aromatic hydrocarbons (HMW-PAHs) are potential threats to health and the environment. They result mainly from incomplete combustion of fossil fuels and organic materials, and they tend to accumulate in terrestrial and aquatic ecosystems (particularly in soils, sediments, and water sources). Chronic exposure to HMW PAHs is associated with some of the most dreadful health outcomes, including lung and skin cancers and disorders of the respiratory and immune systems. The study therefore proposes microbial degradation as a promising bioremediation technique for HMW PAHs: pyrene, benzo[a]pyrene, chrysene, and fluoranthene. Aerobic degradations mediated by dioxygenase and dehydrogenase enzymes, as well as anaerobic pathways involving sulfate- and nitrate-reducing bacteria, are discussed. Factors that promote microbial degradation include pH, temperature, nutrient availability, and salinity. While all factors can be biostimulation and bioaugmentation, the study emphasizes these two as effective methods to enhance bioavailability and degradation efficiency. The results provide insightful information for further development of microbial techniques in remediation of HMW PAH-contaminated sites.

## Introduction

**Fig. 1:**
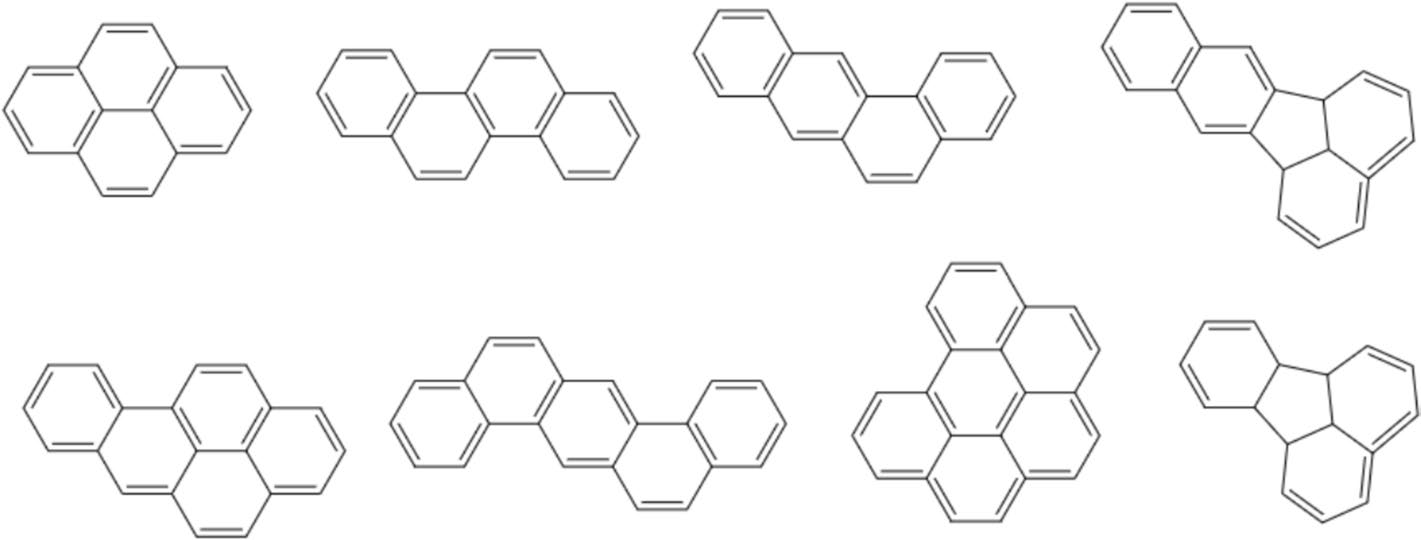
HMW PAHs listed as priority pollutants by US EPA

Polycyclic aromatic hydrocarbons (PAHs) consisting of carbon and hydrogen atoms are common organic pollutants found in the environment, and they are classified into low molecular weight PAH (LMW PAHs) with less than four rings and high molecular weight PAH (HMW PAHs) with four rings or more than four rings under the listing by the US Environmental Protection Agency (US EPA), as shown in Fig. 1.

The main source of PAHs is the incomplete combustion of fossil fuels and petroleum products. Concerns are raised on the persistence of the PAH pollutants in the environment including soil, marine water and surface water, and air due to the genotoxicity and carcinogenicity of the PAH pollutants especially HMW PAH. The toxicity of PAHs, most importantly the HMW compounds, is greatly concerning from an environmental perspective. Most of the PAHs are genotoxic compounds, which indicate that they cause genetic mutations due to interaction with cellular DNA. Hence, these compounds increase cancer and other pathologies risks in all living organisms exposed to them. The highly carcinogenic attributes of some PAHs, like benzo[a]pyrene, have been long established in scientific literature; therefore, they remain a significant concern regarding environmental safety as well as public health [1]. The persistence and the low bioavailability of the HMW PAH are contributed by the sorption of HMW PAH compounds to the soils and sediments, added that they are hydrophobes, leading to the lower biodegradability of HMW PAHs compared to LMW PAHs. There are several existing remediation methods of PAH-contaminated sites including incineration and in situ thermal desorption (ISTD), solvent extraction, and chemical oxidation [1, 2]. However chemical methods like chemical oxidation can induce a higher cost of treatment and possibly cause secondary contamination to the soils due to usage of chemical agents. The pollutants are not destroyed but only transferred to other places through physical treatment such as solvent extraction, and the extracts still require secondary treatment [2]. The ISTD method requires higher energy; hence, a higher cost is induced from the installed off-gas control devices of the incinerator [3]. Bioremediation becomes a promising remediation method of PAHs and is favored due to its eco-friendliness. The destruction of PAH pollutants can be achieved through the transformation of hazardous pollutants into non-hazardous products by microorganisms during bioremediation. Extensive studies have been covered on the bacterial degradation of LMW PAHs but not HMW PAHs. However, there is limited knowledge about the specific role and interactions within microbial consortia that enhance the biodegradation of HMW PAHs. The mechanisms of aerobic and anaerobic degradation pathways of HMW PAHs are not fully understood. Thus, the aim of this study was on the microbial degradation of HMW PAHs by the bacteria including aerobic and anaerobic mechanisms and metabolic pathways of PAHs. The utilization of microbial consortium in degrading PAHs is also reviewed in this study. The bioremediation and degradation of PAHs by bacteria are often limited by various abiotic factors including temperature, pH, and nutrients, and these factors affecting PAH degradation are discussed in this study.

## Source and Occurrence of PAH

**Fig. 2:**
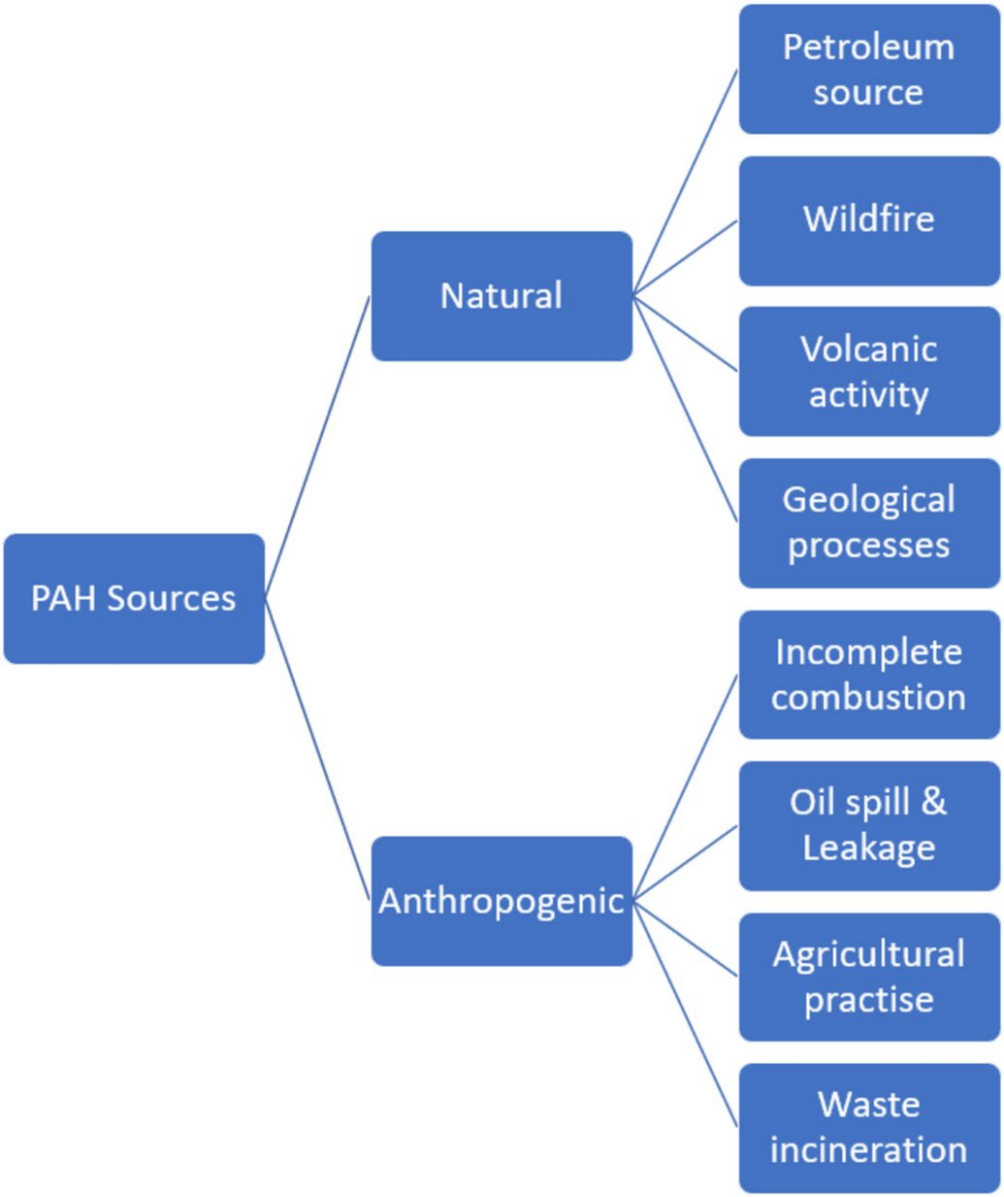
Natural and anthropogenic sources of PAHs

Polycyclic aromatic hydrocarbons have sources that can be grouped into petrogenic, pyrogenic, and biological origins. The pyrolysis of organic materials such as coal, wood, and petroleum products under high-temperature conditions and in the absence of oxygen is responsible for the development of pyrogenic PAHs. Frequently, these PAHs are emitted during the incomplete combustion of wood products, vehicle fuels, and fuel oil. Typically, low molecular weight PAHs are dominant for pyrogenic PAHs and linked to combustion processes. Petrogenic PAHs, on the other hand, are those that emanate from petroleum and its derivatives. These origins explain their formation as a natural process over geological time involving maturation of organic matter under heat and pressure. High molecular weight PAHs are more commonly observed in petrogenic PAHs and therefore relate to production, transport, and usage of fossil fuels (oil and gas). Contamination of the environment by petrogenic PAHs is usual in cases where there has been accidental oil spillage, leakage from storage tanks, or the release of hydrocarbons during extraction or refining processes. These are important contributors to PAH pollution in marine and terrestrial environments. The synthesis of biological PAH by certain microorganisms and plants is another source of PAHs. Apart from the degradation of vegetations and petroleum source rock erosion, the eruptions of volcanoes and forest fires related to incomplete combustion are classified as the natural sources of PAHs. The incomplete combustion process of the industries and emission of the vehicles and aircraft are some of the anthropogenic sources of PAHs. Industrial emissions are sourced from the manufacturing plant including iron and steel, cement, and dye, as well as the diesel engines and industrial furnace [1, 4, 5]. The natural and anthropogenic sources of PAHs are shown in Fig. 2.

The primary source of PAH in the environment is the atmospheric emission when organic materials are combusted incompletely. There is a higher concentration level of PAHs in urban areas due to the emission from industrial plants and vehicles. As the high molecular weight of PAH is related to their low vapor pressure, HMW PAH with lower vapor pressure like benzo(a)pyrene tends to have greater sorption to the particles in the atmosphere compared to LMW PAH. As result, HMW PAHs are more concentrated in the vapor phase compared to the solid phase in the atmosphere. These emitted PAH in the air will eventually end up in the terrestrial environment by dry or wet deposition in the soils and sediments, while the deposited PAHs mostly undergo sorption to the soil [4]. The PAH in the soils from landfill leachate or irrigation can further migrate to the groundwater causing groundwater contamination [6]. Natural oil seeps are the major contributor to the occurrence of PAH in marine water, added by other contributors including accidental oil spill and wastewater effluents containing PAHs. The permanent settling of the PAH pollutants in the marine sediments is caused by hydrophobicity and low aqueous solubility [7]. The occurrence of PAHs in surface water originates from atmospheric deposition of PAHs and the discharge of industrial effluents and wastewater as well as surface runoff. The contamination of the surface water bodies by PAHs leads to the occurrence of PAHs in drinking water, posing human health risk through consumption of drinking water. PAHs found in the crops and vegetables are sourced from two pathways, which are the plant uptake of PAH pollutants through contaminated soils and the deposition of atmospheric PAH pollutants on the surface of vegetables and fruits especially those with waxy and large leaves. In addition, grilled and smoked meats and seafood as well as beverages also contain some PAHs [7].

The case study presented below is the contamination of sediments at Johor Strait, Malaysia, by PAH. The study results revealed the occurrence of moderate PAH pollution based on the concentration level. The PAHs found in the contaminated sediments are dominated by high molecular weight PAHs. The highest percentage of PAHs discovered in the sediments is 35% of four rings PAHs, while both five rings and six rings PAHs accounted for 13%, respectively. The main source of PAHs is from the pyrogenic source, which is associated with incomplete combustion of organic matter. Therefore, it is speculated that these PAHs in the sediments are sourced from the release of atmospheric PAH pollutants due to the uncontrolled burning of biomass occurring in Sumatra, Indonesia. Apart from that, other sources of PAHs in the sediments are also identified including vehicle exhausts, combustion of coal at coal power plants, and petroleum products, which are also known as petrogenic sources [8]. In the case of the Shanxi chemical plant in China, heavy contamination of soils by PAHs occur according to the PAH concentration. The dominance of HMW PAHs in the sampled soils is illustrated by 34.9% of five rings PAHs and 16% of four rings PAHs; hence, the identified source of PAHs is emission from heavy traffic and the combustion of coal and wood at the industrial areas. The lower percentage of LMW PAHs in soils is attributed to their greater biodegradability. The vertical distribution profile of PAHs in soils shows the decline in the PAH concentration with the increase in depth [9]. Table 1 presents the occurrence of PAHs in the terrestrial and marine environment in different countries.

**Table 1:** Occurrence of PAHs in different countries

Countries	PAHs found	Sources	References
Malaysia	Naphthalene, Phenanthrene, Anthracene, Acenaphthene, Acenaphthylene, Fluorene, Fluoranthene, Pyrene, Benzo[k]fluoranthene, Benzo[e]pyrene, Indeno[1,2,3-cd]pyrene, and Benzo[ghi]perylene	River and estuarial sediments	[10]
	Naphthalene, Phenanthrene, Anthracene, Acenaphthene, Acenaphthylene, Fluorene, Fluoranthene, Pyrene, Chrysene, Benzo[b]fluoranthene, Perylene, Benzo[k]fluoranthene, Indeno[1,2,3-cd]pyrene, Benzo[ghi]perylene, and Benzo[a]anthracene	Marine sediments	[11]
Indonesia	Naphthalene, Phenanthrene, Anthracene, Acenaphthene, Acenaphthylene, Fluorene, Fluoranthene, Pyrene, Chrysene, Benzo[a]anthracene, Benzo[b]fluoranthene, Benzo[k]fluoranthene, Benzo[a]pyrene, Indeno[1,2,3-cd]pyrene, Dibenzo[a,h]anthracene, and Benzo[ghi]perylene	River sediments	[12]
Denmark	Fluorene, Fluoranthene, Benzo[k]fluoranthene, Benzo[b]fluoranthene, Benzo[a]pyrene, and Benzo[ghi]perylene	soils	[13]
Korea	Phenanthrene, Anthracene, Fluoranthene, Pyrene, Chrysene, Benzo[a]anthracene, Benzo[b]fluoranthene, and Benzo[k]fluoranthene	Paddy soils	[14]
Iran	Naphthalene, Acenaphthylene, Acenaphthene, Phenanthrene, Chrysene, Benzo[a]anthracene, Benzo[b]fluoranthene, Benzo[k]fluoranthene, Benzo[a]pyrene, Indeno[1,2,3-cd]pyrene, Dibenzo[a,h]anthracene, and Benzo[ghi]perylene	Drinking water	[15]
Nigeria	Naphthalene, Acenaphthene, Acenaphthylene, Fluoranthene, Anthracene, Fluorene, and Phenanthrene	Drinking water	[16]
Italy	Naphthalene, Acenaphthene, Acenaphthylene, Fluoranthene, Anthracene, Fluorene, and Phenanthrene	Marine sediments	[17]
Brazil	Naphthalene, Fluorene, Phenanthrene, Anthracene, Fluoranthene, Pyrene, Chrysene, Acenaphthylene, and Acenaphthene	Marine sediments	[18]

The results are consistent with global trends in PAH pollution, by noting the persistence of HMW PAHs in industrial and urban locations. They underscore microbial biodegradation as a sustainable remediation strategy, complementing efforts to reduce environmental cleanup. Improvement of bioremediation techniques advances global goals in the reduction of PAH impacts on ecosystems and human health.

## Impact of PAH on Human Health and Environment

**Fig. 3:**
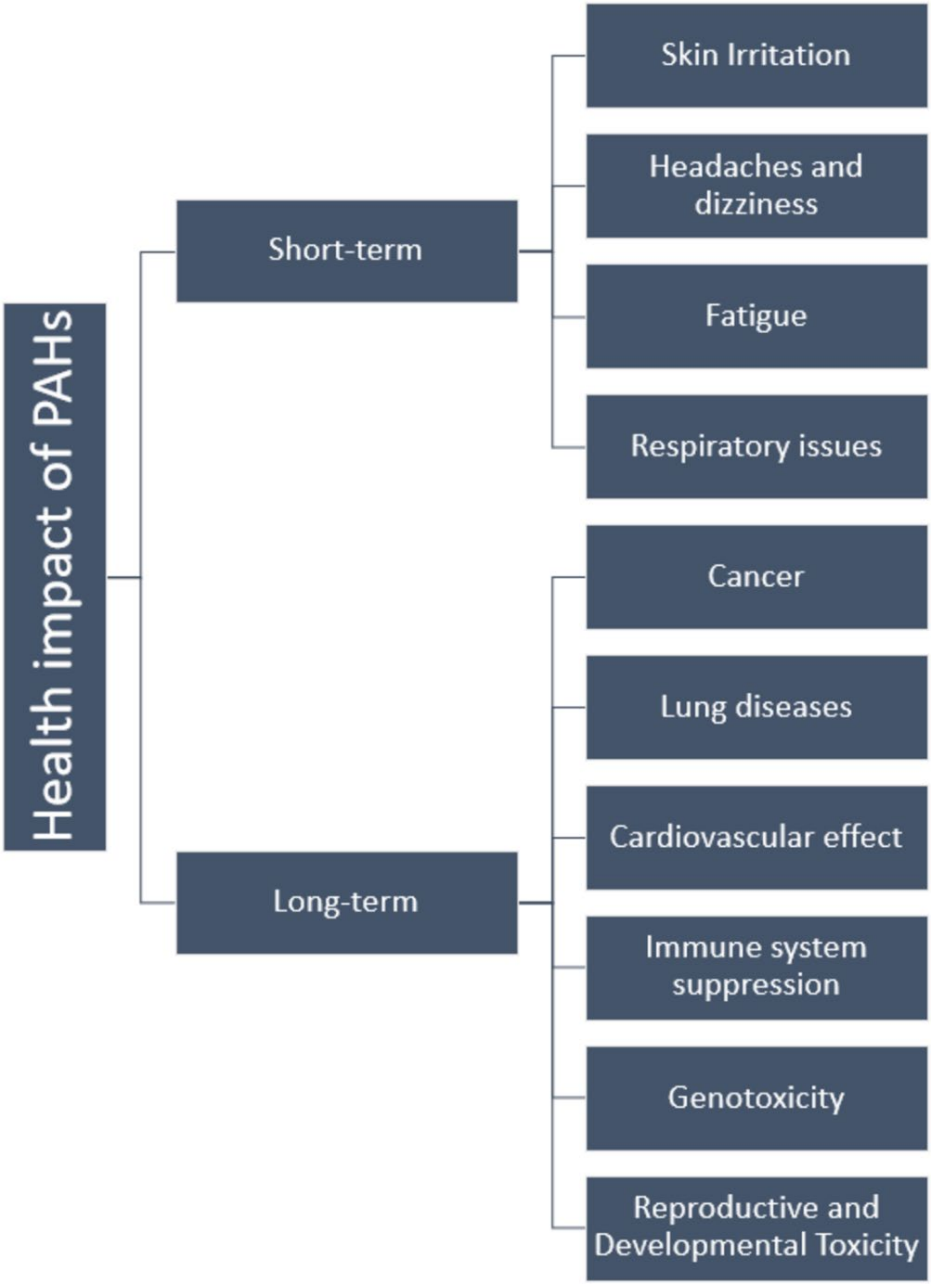
Health impacts of PAH on human

Human exposure to PAHs occurs through many pathways, such as inhalation of air contaminated with PAHs, dermal absorption from touching contaminated soil, water, or sediment, and dietary intake of food contaminated with PAHs, particularly grilled or smoked foods. Since PAHs can bioaccumulate in food chains, higher trophic level organisms (humans included) receive a greater concentration of these toxic compounds. Health effects of PAHs—genotoxicity and carcinogenicity—are highly dependent on the level of exposure and the specific PAH involved. These pose significant risks to human health. Skin allergies and irritation are one of the acute health impacts with exposure to PAH mixture including benzo[a]pyrene and anthracene. The occurrence of lung cancer and respiratory problems from the inhalation of PAHs is another chronic health impact of PAHs. Apart from cataract, damage to the liver and kidney can occur from exposure to PAH (long term). Birth defects and problems with reproductive system and hormones are the consequences of PAH exposure [4, 19]. Workers are normally under heavy exposure to PAH pollutants while working in the industries like coking plant and power plant. Higher cancer risk including gastrointestinal and skin cancer and problems with the immune system are the common health issue found in the workers working in the industries undergoing long-term PAH exposure and exposure to other pollutants. Other health problems associated with the respiratory system of workers include lung cancer, bronchitis, and possibly asthma [4, 20]. The carcinogenicity of the PAH is caused b7 the binding ability of the metabolites to the DNA hence resulting in the occurrence of cancer and possible mutation. HMW PAHs remain in ecosystems and may therefore lead to ecological risk. In aquatic environments, they bioaccumulate in living organisms, disturb reproduction processes, and damage early stages of development; all this has a negative effect on biodiversity. Changed microbial diversity in soils makes HMW PAHs less damaging, reducing beneficial microbes and increasing the more tolerant strains to pollutants. The nutrient cycling, health of the soils, and stability of the ecosystems are distressingly imbalanced towards malremediation. Figure 3 summarizes the health impact of PAHs on human. The bioaccumulation of PAH compounds in marine or aquatic species can occur due to increased concentration of PAH and increased toxicity, from the intake of PAH through ingestion and skin contact. Severe soil contamination by PAHs can exert toxicity on the terrestrial invertebrates. The accumulation of PAHs in filtering organisms can occur as they are less capable of PAH metabolism, coupled with the exceeding level of PAH concentration from wastewater discharge [4].

## Microbial Degradation of PAH by Bacteria

### Aerobic Degradation

**Fig. 4:**
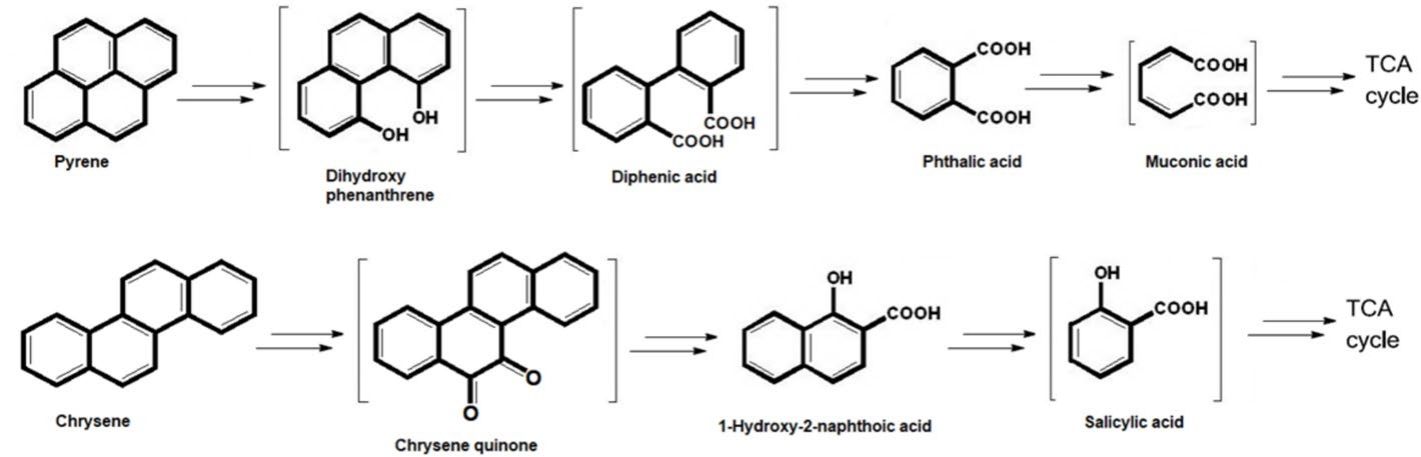
Biodegradation of PAHs by aerobic bacteria [22]

The bioremediation of organic pollutants including PAH compounds through bacterial degradation is governed by the enzymes present in the bacteria. Under the group of oxidoreductases, oxygenase enzymes including monooxygenase and dioxygenase are significant for the degradation of PAH and hydrocarbon compounds by bacteria through metabolism. Oxygen acts as the final electron acceptor and co-substrate [21]. As shown in Fig. 4, metabolic pathway of pyrene degradation starts with its transformation by dioxygenases, which leads to the formation of dihydroxyphenanthrene and 2,2′-diphenic acid. From these intermediates, phthalic acid is formed, which can be further degraded by hydroxylation and cleavage reactions to mucronic acid that enters TCA cycle. Key enzymes involved in these pathways include dioxygenases for aromatic ring cleavage, diohydrodiol dehydrogenase involved in the conversion of dihydroxyphenanthrene, and other ring-cleaving enzymes involved in converting phthalic acid to mucronic acid. The culture of *Hortaea* sp. B15, mucronic acid, diphenic acid, and dihydroxyphenanthrene were not detected; therefore, it suggests either pathways are not complete under the conditions studied or unrecognized. Chrysene degradation pathway has been investigated. The very first step involves the action of dioxygenases, which cleave the aromatic rings leading to the formation of chrysenequinone from chrysene. This step is more changed into 1-hydroxy-2-naphthoic acid, which sees more enzyme change likely from hydroxylases and dehydrogenases to make salicylic acid. Salicylic acid then gets turned into steps that go into the tricarboxylic acid (TCA) cycle finishing off the breakdown path. But, chrysenequinone and salicylic acid were not seen in the grow of Hortaea sp. B15, hinting either that these things got broke down fast or that other break paths might be happening under the test rules [22].

**Fig. 5:**
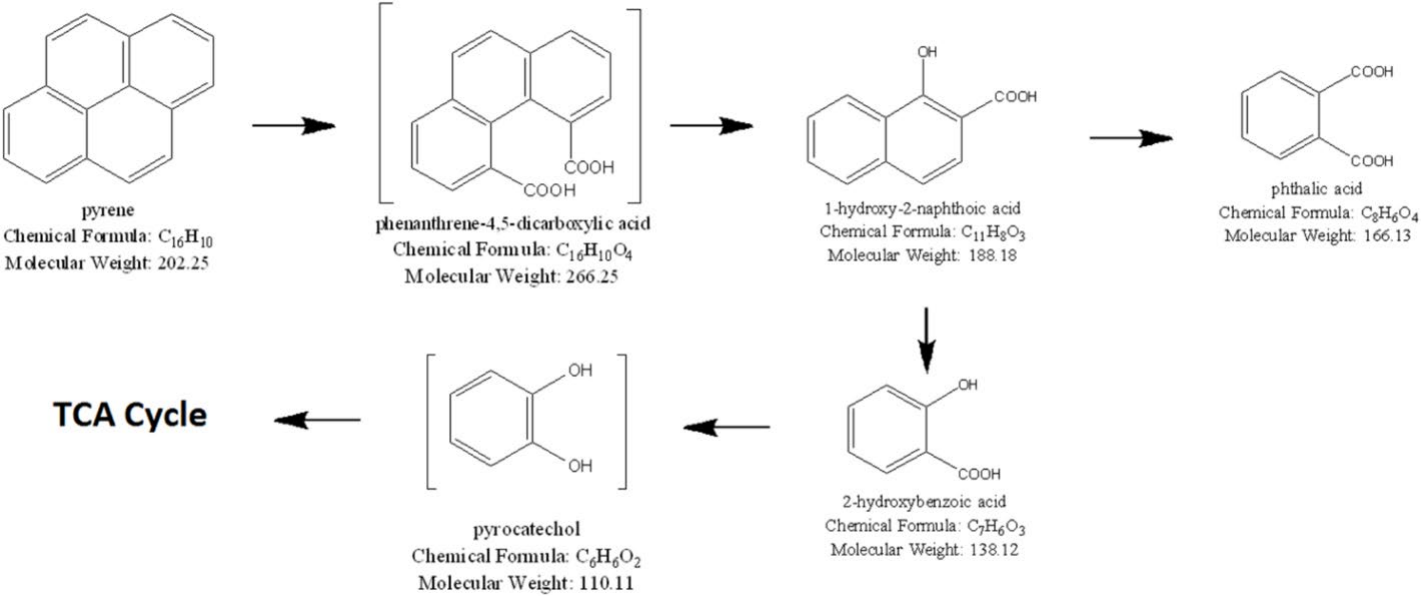
Proposed pathway of pyrene degradation by *Coriolopsis caperata* BM-172, *Fomes fomentarius* BM-745, and *Pluteus chysophaeus* BM-792 [25]

The metabolic pathway of PAH degradation by bacteria is being reviewed by Seo et al. and Peng et al. [23, 24]. Table 2 shows the studies on HMW PAH degradation by different bacteria strains such as Bacillus, Mycobacterium, and Pseudomonas, isolated from the PAH-contaminated sites. Previous study investigated that *Coriolopsis caperata* BM-172, *Fomes fomentarius* BM-745, and *Pluteus chysophaeus* BM-792 transformed pyrene to 1-hydrox-2-naphtoic acid, 2-hydroxybenzoic acid, and phthalic acid by laccase system (Fig. 5) [25]. From the study by Nzila et al., the isolated strain of *Achromobacter xylosoxidans* PY4 is utilized in the degradation of pyrene and the metabolites identified include dibutyl-phthalate and monohydroxypyrene. The optimum condition for bacterial growth and pyrene degradation is pH value of 8 and 37 $^\circ{\rm C}$. Phenanthrene is formed under the oxidation of pyrene with one oxygen atom where monooxygenase acts as catalyst [26].

**Table 2:** Degradation of HMW PAHs by various bacteria

PAHs	Mechanism	Bacteria	Source
Pyrene	Aerobic	*Burkholderia fungorum*, *Caulobacter* sp.	Oil contaminated site
	Aerobic	*Cycloclasticus* sp. P1	Deep sea sediments
	Aerobic	*Bacillus pumilus*	Soil contaminated by crude oil
	Aerobic	*Bacillus* sp. C7	Coal deposited soil
	Aerobic	*Herbaspirillum chlorophenolicum* strain FA1	Activated sludge of wastewater treatment plant
	Aerobic	*Achromobacter denitrificans* ASU-035	Oil contaminated soil
	Aerobic	*Mycobacterium gilvum* CP13	Activated sludge
	Aerobic	*Pseudomonas* sp. ISTPY2	Contaminated landfill soil
Benzo[a]pyrene	Aerobic	*Bacillus subtilis* BMT4i	Automobile contaminated soil
	Aerobic	*Sphingomonas yanoikuyae* JAR02	Contaminated soil
	consortium	*Pseudomonas* sp.	Contaminated sediment of
	Aerobic	*Brevibacillus brevis*, *Bacillus licheniformis* M2-7	E-waste contaminated area
	Aerobic	*Mesoflavibacter zeaxanthinifaciens*, *Mesorhizobium septentrionale*, *Mycobacterium fluoranthenivorans*, *Bacillus rnegaterium*, *Mycobacterium brisbanense*, *Sphingobium amiense*, *Olleya* sp., *Vibrio rumoiensis*, *Herbiconiux ginsengi*	Contaminated water and mud
Chrysene	Aerobic	*Pseudoxanthomonas* sp. PNK-04	Soil contaminated by coal
	Aerobic	*Bacillus halotolerans*	Oil-contaminated water
	Aerobic	*Paracoccus aminovorans*	Activated sludge from coking plant
Fluoranthene	Consortium	*Pseudomonas aeruginosa* PSA5, *Rhodococcus* sp. NJ2	Petroleum sludge
	Aerobic	*Herbaspirillum chlorophenolicum* FA1	Activated sludge
	Aerobic	*Bacillus circulans*, *Alcaligenes faecalis*, *Enterobacter*, *Listeria*, *Staphylococcus*, *Celeribacter indicus* P73	Contaminated mangrove sediments
	Consortium	*Cycloclasticus* NY93E, PY97M, PY97 N	Yellow sea sediments
	Aerobic	*Pseudomonas aeruginosa* DN1	Soil contaminated by petroleum

Based on the study by Zhang et al., 97.7% of pyrene degradation by *Janibacter anopheles*’ strain JY11 is achieved at 30 $^\circ{\rm C}$ and neutral pH. The decline in the PAH degradation rate is observed when there is high PAH concentration due to elevated toxicity induced by the metabolites formed or low PAH concentration due to reduced carbon source in supporting bacterial growth. In the case of this study, simultaneous degradation of PAH mixture causes declining degradation rate with the inhibition by the substrates and high PAH concentration [27]. Based on the study by Nzila et al., *Staphylococcus haemoliticus* strain 10SBZ1 A can perform optimum benzo[a]pyrene degradation at neutral pH, 10% of sodium chloride and 37 $^\circ{\rm C}$, as well as the degradation of PAH compounds with lower molecular weight [28]. The study by Arulazhagan and Vasudevan investigated PAH degradation utilizing *Ochrobactrum* sp. VA1, which is isolated from the microbial consortium enriched by contaminated marine water. 84% of pyrene degradation with 57% of benzo[k]fluoranthene and 50% of benzo(e)pyrene under different period of days are reported in the study. The addition of yeast extract is required at increased NaCl concentration till 60 g/L due to the decline in PAH degradation rate under high salinity conditions. PAH degradation including benzo(e)pyrene and fluorene by *Ochrobactrum* sp. VA1 in petroleum wastewater is facilitated through the utilization of added nutrients, which are nitrogen and phosphate by the strain. The halotolerance of this bacterial strain is suitable for the degradation of PAH in the polluted marine environment [29]. Based on the study by Mishra and Singh on Bap degradation by *Rhodococcus* sp. NJ2 and *Pseudomonas aeruginosa* PSA5, one of the induced enzymes is salicylate hydroxylase, which is associated with the transformation of salicylate to catechol involving binding of molecular oxygen with substrate complex. Catechol 1,2-dioxygenase is involved in the ring oxidation of PAH compound through the incorporation of oxygen atoms while other enzymes identified during Bap degradation are 2-carboxybenzaldehyde dehydrogenase and Catechol 2,3-dioxygenase [30].

PAHs become more hydrophobic and electrochemically stable with increased aromatic ring number and angularity, causing the reduction in the bioavailability of PAHs and enhancing the sorption of PAHs to soil. The low aqueous solubility of PAHs affects the bioavailability of PAHs and microbial degradation. Biosurfactants can play an important role in improving the PAH bioavailability through reducing surface tension and increasing substrate surface area. One of the mechanisms of PAH desorption by biosurfactants is micellar solubilization. The PAH transport to the water phase is facilitated by the solubilization of PAH compounds at the hydrophobic core of micelle formed by the biosurfactant. The contaminant matrix is modified by the biosurfactants through increasing the interfacial area of soils and the diffusivity of PAHs. The bacterial adherence to PAHs can also be enhanced by the alteration of cell surface induced by the biosurfactants [31, 32]. The study by Dhote et al. studied the chrysene biodegradation by biosurfactant-producing bacteria, which are *Pseudomonas* sp. and *Bacillus* sp. isolated from the oily sludge. The emulsification of PAH compounds by biosurfactant can increase the bioavailability of PAH compounds due to the reduced surface tension and increased solubility by biosurfactant. The meta cleavage degradation pathway of chrysene is speculated based on the identified gene of catechol 2.3 dioxygenase [33]. Bezza and Chirwa demonstrated significant reduction and degradation of PAH under the soil amended by biosurfactant, which is lipopeptide produced from *Bacillus cereus* SPL-4 in the microcosm. The dissipation rate of chrysene, fluoranthene, and pyrene are 82%, 65%. and 62%, respectively [34]. With the isolation of bacteria from petroleum sludge, Mishra and Singh investigated the benzo[a]pyrene degradation by *Rhodococcus* sp. NJ2 and *Pseudomonas aeruginosa* PSA5 and the results show respective degradation rate of 47% and 88% after incubation for 25 days. The production of glycolipid, which is known as biosurfactant by both bacteria, facilitates the degradation of benzo[a]pyrene due to high emulsification activity and reduction in water surface tension [30]. These studies on aerobic biodegradation of HMW PAHs by different bacteria are summarized in Table 3.

**Table 3:** Research on aerobic biodegradation of HMW PAHs

HMW PAH compounds	Bacteria	Location	References
Pyrene	*Achromobacter xylosoxidans* PY4	Contaminated soil	[26]
Pyrene	*Janibacter anopheles* strain JY11	Contaminated soil	[27]
BaP	*Staphylococcus haemoliticus* strain 10SBZ1 A	Contaminated soil	[28]
Pyrene, benzo[k]fluoranthene and BeP	*Ochrobactrum* sp. VA1	Marine water contaminated by petroleum or coal	[29]
BaP	*Rhodococcus* sp. NJ2 and *Pseudomonas aeruginosa* PSA5	Oily sludge	[30]
Chrysene	*Pseudomonas* sp. and *Bacillus* sp.	Oily sludge	[33]
chrysene, fluoranthene, and pyrene	*Bacillus cereus* SPL-4	Contaminated soil	[32]

### Microbial Consortium

Bacterial communities in the degradation of PAH pollutants provide an efficient environmental remediation technique. Microbial consortia, made up of different PAH-degrading microbes, can attack a broader spectrum of PAH compounds and much higher rates of degradation. Microbial consortia display greater metabolic diversity as compared to pure cultures; hence, robust and efficient PAH degradation under a wide variety of conditions [34]. With the enrichment by PAH-polluted soils, the bacterial community is utilized for degradation of PAHs in soils in the study by Lu et al. There are 35.4% and 52.0% PAH removal rate of HMW PAH and LMW PAH, respectively, and the degradation of all PAH types involved is not inhibited due to the bacterial diversity. The bacteria involved in HMW PAH degradation is speculated to include Nocardioides and Actinobacteria as there is an increased degradation rate of HMW PAH upon their presence as major strains [35]. Vaidya et al. studied the chrysene degradation of the bacterial consortium ASDC developed from the contaminated soil and sediments and reported the existence of *Bacillus* sp., *Rhodococcus* sp., and *Burkholderia* sp. It is observed that the chrysene degradation correlates with the bacterial consortium and excess concentration level of chrysene causes inhibition to the degradation of chrysene and bacterial growth simultaneously [36]. Another study by Vaidya et al. focused on the pyrene degradation by bacterial consortium consisting of *Rhodococcus* sp. ASDP3, *Burkholderia* sp. ASDP2, and *Pseudomonas* sp. ASDP1 developed from the contaminated sediments [37]. In both studies, the enhanced degradation rate of chrysene and pyrene along with other PAH compounds is reported due to co-metabolism with increased carbon source where simultaneous degradation of PAH compounds having similar structures occurs. The optimum degradation conditions are neutral pH and 37 $^\circ{\rm C}$ and both chrysene and pyrene degradation occur through the phthalic acid pathway. Improved degradation of chrysene and pyrene with other PAH compounds presented at the microcosm is also demonstrated in both studies where there is 99% of pyrene degradation by the consortium in microcosm, with the assistance of the indigenous microorganisms in performing efficient degradation [36, 37]. Sun et al. studied the degradation of HMW PAH by the bacterial consortium of polluted soil from Beijing Coking Chemical Plant. It is observed that the degradation ability and performance of pyrene, benzo(a)pyrene, and fluoranthene is great from the results. The consortium is found to be dominated by the proteobacteria group including alphaproteobacteria, betaproteobacteria, and gammaproteobacteria, implying that they are the main degrader of HMW PAH compounds [38]. The study conducted by Vila et al. analyzed the PAH degradation involving the inoculation of marine microbial consortium with sand contaminated by Prestige fuel oil. It is concluded that there is 47 to 80% degradation of alkyl chrysenes, while the degradation of alkyl fluoranthene or pyrene is around 49 to 60%. Gamma-proteobacteria (*Methylophaga and Marinobacter*) is identified as the microbes associated with the degradation of HMW PAH contained in the fuel oil including chrysene, benzo(a)anthracene, and fluoranthene. *Alphaproteobacteria* and *Gordonia* degrade the pyrene compound, as proven by the detection of these bacteria in the pyrene culture [39]. Another study by Gallego et al. focus on the pyrene-degrading consortium with inoculation of fuel oil-contaminated beach sand, and the results showed that 34% and 75% of pyrene removal occurs. The 31% mineralization of pyrene occurs under the concerted efforts by various microbial components as there is absence of partially oxidized intermediates. Based on the gene analysis, the presence of the representatives of Gordonia in degrading pyrene is implied due to the detection of DNA sequence in close relation to the NidA3 dioxygenase of mycobacterial strain acting as pyrene degrader [40]. The degradation of HMW PAH contained in the fuel oil by *Mycobacterium* sp. strain AP1 is investigated in the study conducted by Vila and Grifoll. The result of the study illustrates 75% reduction of pyrene, 80% reduction of fluoranthene, and only 30% reduction of benzo(a)anthracene, while pyrene and fluoranthene promote the growth of bacteria when degradation by mycobacterium occurs at the same time. The alkyl substitutes of the PAH compounds, which are monomethylated pyrene or fluoranthene, also can be degraded by the strain AP1 [41]. The studies on HMW PAH degradation by microbial consortium are listed in Table 4.

**Table 4:** Biodegradation of HMW PAHs by various microbial consortium

HMW PAH compounds	Bacteria in microbial consortium	Source	References
Mixed HMW PAHs	Nocardioides and actinobacteria	Contaminated soils	[35]
Chrysene	*Bacillus* sp., *Rhodococcus* sp., and *Burkholderia* sp.	Contaminated sand sediments	[36]
Pyrene	*Rhodococcus* sp. ASDP3, Burkholderia sp. ASDP2 and *Pseudomonas* sp. ASDP1	Contaminated sand sediments	[37]
Pyrene, benzo(a)pyrene, and fluoranthene	Alphaproteobacteria, betaproteobacteria and gammaproteobacteria	Contaminated soil	[38]
Chrysene, benzo(a)anthracene, and fluoranthene	Gamma-proteobacteria (*Marinobacter* and *Methylophaga*)	beach sand contaminated by fuel oil	[39]
Pyrene	*Alphaproteobacteria* and *Gordonia*	beach sand contaminated by fuel oil	[40]
Pyrene	Gordonia	beach sand contaminated by fuel oil	[40]
Pyrene, fluoranthene, and benzo[a]anthracene	*Mycobacterium* sp. strain AP1	Seawater contaminated by fuel oil	[41]

### Anaerobic Degradation

**Fig. 6:**
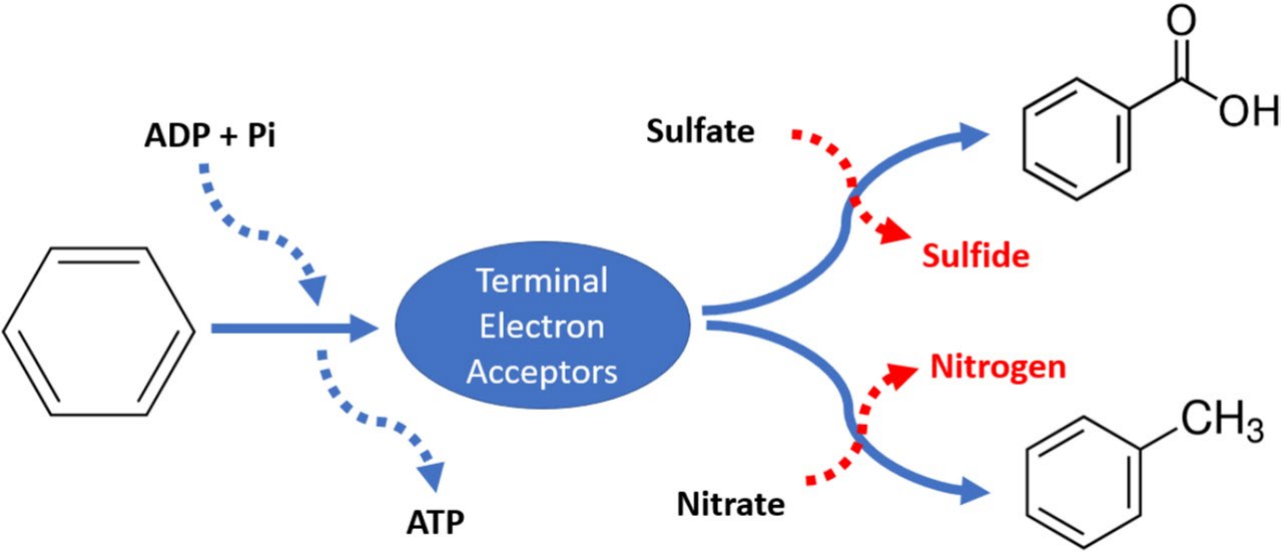
Anaerobic biodegradation of PAHs

Anaerobic degradation of high molecular weight PAHs involves nitrate, sulfate, or iron ions as terminal electron acceptors in the absence of oxygen. In this process, electrons released in the primary cleavage of the PAH molecule can be transferred to these electron acceptors, which permit functioning of the microbial respiratory chain under anoxic conditions. This electron transfer process generates energy for microbial growth by converting adenosine diphosphate into adenosine triphosphate. Nitrate-reducing bacteria, sulfate-reducing bacteria, and iron-reducing bacteria are the dominant microorganisms involved in this pathway for electron transport that aids in anaerobic metabolism for the degradation of PAHs in low-oxygen environments such as sediments, groundwater, and anoxic soils (Fig. 6).

The transformation of these terminal electron acceptors into nitrogen or sulfide occurs is facilitated by bacteria such as denitrifying bacteria or sulfate-reducing bacteria [42]. There are relatively few studies on the anaerobic degradation of HMW PAHs compared to the studies on the HMW PAHs aerobic degradation. The issue with the anaerobic degradation of PAHs is the tendency of PAH having higher stability of their carbon–carbon bond and carbon-hydrogen bond upon anoxic conditions [43]. However, the PAH compounds often exist in the environment under anoxic conditions like in the soil layer. The advantages of anaerobic microbial degradation are the decrease in the available nitrates and sulfate apart from the reduction in PAH compounds.

Yan et al. studied the anaerobic degradation of pyrene and benzo[a]pyrene involving *Hydrogenophaga* sp. PYR1 isolated from the sediments polluted by PAH. The benzo[a]pyrene anaerobic degradation under iron-reducing conditions is discovered, as there is accumulation of Fe^2+^ indicating the occurrence of iron reduction where the terminal electron acceptor is ferric citrate. The production of biosurfactant by the bacterial strain stimulated by the iron can increase the PAH bioavailability, improving the degradation of pyrene and benzo[a]pyrene. The produced 5-ethylchrysene from initial degradation of benzo[a]pyrene is converted into pyrene, then phenanthrene and 1H-Phenalen-1-one is generated from pyrene [43]. Yang et al. investigated the anaerobic biodegradation of pyrene under denitrification conditions where reduction of nitrate and nitrite occurs during pyrene degradation by *Paracoccus denitrificans* isolated from sediments polluted by petroleum. High nitrate concentration can induce the activity of nitrate reductase, causing accumulation of nitrite. The induction of nitrite reduction at high NO_2_^−^ concentration prior to high activity of nitrate reductase allows complete denitrification hence improving pyrene degradation efficiency.

Low pyrene degradation efficiency is observed at high initial pyrene concentration due to inhibition caused by intermediate products of degradation. Increased C/N ratio can cause increase in pyrene degradation efficiency with more carbon sources for *Paracoccus denitrificans*. The study concluded that pyrene degradation efficiency under denitrification condition can be enhanced at low concentrations of nitrate and nitrite and high C/N ratio [44]. The identified bacterial strain for anaerobic degradation of pyrene is *Klebsiella* sp. LZ6 in the study conducted by Li et al. Under 10% inoculum ratio, 33% of the degradation rate of pyrene is achieved. Optimal temperature for degradation of pyrene by the strain is 30 °C, and the most effective degradation occurs within a pH range of 6.0 to 8.4. However, as the concentration of pyrene increases the rate of degradation observed declines, possibly because of substrate inhibition or limited microbial metabolic capacity to process higher concentrations of pyrene. Pyrene,4,5-dihydro is formed after pyrene undergoes hydrogenation reduction, then phenanthrene is produced through carbon–carbon bond cleavage [45].

The isolation of *Pseudomonas* sp. JP1 from Shantou Bay and the anaerobic degradation of benzo[a]pyrene and other LMW PAH are investigated in the study by Liang et al. The degradation rate of Bap under anaerobic conditions is around 30%, and the degradation is facilitated by the denitrifying bacteria, which is *Pseudomonas*. Acting as the terminal electron acceptor during the degradation, denitrification occurs where nitrate is transformed into nitrite involving different reductases such as nitrite reductase and nitrate reductase [46]. In the study by Li et al., enhanced reducing conditions at a greater depth are suggested due to the observed reduction of the concentration of electron acceptors including nitrate and Fe^3+^ ions with increasing depth of the sediments, and the available electron acceptors in the sediments are dominated by sulfate ions. The bacteria degrading mixed PAH containing pyrene and fluoranthene under low oxygen conditions include *Rhodococcus*, *Microbacterium*, and *Sphingonomas* while only *Sphingonomas* is identified from the PAH degradation with the absence of oxygen [47]. Under nitrate-reducing conditions, Qin et al. identified *Cellulosimicrobium cellulans* CWS2 isolated from the polluted soil as the degrader for benzo[a]pyrene. High degradation rate of benzo[a]pyrene is demonstrated with temperature exceeding 30 $^\circ{\rm C}$ and alkaline conditions where the highest degradation rate within 13 days is reported, which is 78.8% at 10 mg/L of Bap concentration. The decreased degradation rate of benzo[a]pyrene is caused by the introduction of strong LMW organic acid, which induces low pH environment as higher degradation efficiency is reported at pH of 7 to 10 but adding a suitable amount of glucose can improve the PAH degradation [48]. Table 5 summarizes the studies on the anaerobic biodegradation of HMW PAHs.

**Table 5:** Research on biodegradation of HMW PAHs by anaerobic bacteria

HMW PAH compounds	Bacteria	Source	References
Pyrene and benzo[a]pyrene	*Hydrogenophaga* sp. PYR1	Contaminated river sediments	[43]
Pyrene	*Paracoccus denitrifica*	Sediments contaminated by petroleum	[44]
pyrene	*Klebsiella* sp. LZ6	Sediments contaminated by crude oil	[45]
Benzo[a]pyrene	*Pseudomonas* sp.	Sediments contaminated by crude oil	[46]
Pyrene and fluoranthene	*Sphingonomas*	Contaminated mangrove sediments	[47]
Benzo[a]pyrene	*Cellulosimicrobium cellulans* CWS2	Contaminated soil	[48]

## Factors Affecting Microbial Degradation of PAHs

The effectiveness of PAH degradation depends on the microbial activity during bioremediation; hence, it is vital to understand what factors affecting the activity of the microorganisms including bacteria, providing a conducive and ideal environment for the microorganism to have their maximum performance in degrading and detoxifying the PAH pollutants. Favorable conditions can promote the growth of bacteria acting as degrader of the PAH pollutants in the environment. The degradation of PAH by the microbes is dependent on the surrounding temperature as temperature has a significant impact on the functionality of the microorganism itself. Mesophilic organisms undergo decreased metabolism due to a reduction in the dissolved oxygen level with increased temperature. Thermophiles can function and perform well under high temperature, while psychrophiles are suited for low-temperature environment [22]. The decline in temperature can cause low substrate affinity hence reducing the sequestration of substrate by the microbes and affect the enzymic activity. The enzymes are denatured, and cell membrane is affected under high temperature, inhibiting the role of microorganisms in degrading PAHs. However increased temperature can lead to increased bioavailability of PAHs due to increased PAH aqueous solubility [6]. The sensitivity of microorganisms towards pH is another factor affecting the microbial activity and degradation of PAH. The optimum pH for the microorganisms as PAH degrader is neutral pH. However, the pH of the contaminated site is often not neutral, affecting the degradation performance by the microorganisms, which favor neutral environment, where the leaching of the construction waste can induce high pH conditions while acidic conditions occur due to the leaching of coal [6, 22]. 35 $^\circ{\rm C}$ and pH value of 7.5 are the optimum condition for the growth of *Planomicrobium alkanoclasticum*, which degrades PAH, as shown in the study by Al-Dossary et al. [49]. Pawar reported that higher PAH degradation is achieved at soil pH of 7.5 as bacterial growth is favored under the same soil pH while higher ATP concentration is also correlated with high PAH degradation rate [50].

Both nutrients and oxygen are the limiting factors of bacterial degradation of PAHs. Some essential nutrients such as phosphorus and nitrogen are required to ensure the normal growth and metabolic activities of the microorganisms. Nutrients can be supplemented and provided at the contaminated sites with low nutrient level to improve the degradation of PAH pollutants through enhancing the metabolic activities of microorganisms. However, inhibiting of microbial degradation can occur with excess level of nutrients [22]. The aerobic degradation of PAHs requires oxygen where the aromatic rings of the PAH molecules are being oxidized by dioxygenase enzymes with introduction of oxygen atoms. Oxygen also acts as electron acceptors and PAH compounds are the electron donors. Apart from that, the production of catabolic enzymes for PAHs degradation is induced by the substrate in the degradation pathway apart from the intermediate products through degradative gene expression. The metabolites can induce more catabolic enzymes of PAHs to enhance the PAHs degradation. On the other hand, the presence of metabolites can compete with the available substrates and even induce toxicity hence inhibiting the PAHs degradation [51]. The metabolites of HMW PAH degradation by different bacteria are shown in Table 6.

**Table 6:** The metabolites of HMW PAH degradation by various bacteria

Bacteria	Mechanism	HMW PAHs	Metabolites	References
*Achromobacter xylosoxidans* PY4	Aerobic	Pyrene	Monohydroxypyrene, 1-methoxyl-2-H-benzo[h]chromene-2-carboxylic acid, 9,10-Phenanthrenequinone, 1-methoxyl-trans-2’-carboxybenzalpyruvate, dibutyl phthalate	[26]
*Staphylococcus haemoliticus* 10SBZ1 A	Aerobic	Benzo[a]pyrene	Dihydroxy-BaP, Bap-quinone, 4,5-chrysene-dicarboxylic acid/4-(8-hydroxypyren-7-yl)−2-oxobut-3-enoic acid/4-(7-hydroxypyren-8-yl)−2-oxobut-3-enoic acid, 10-oxabenzo[def]chrysene-9-one/7-oxabenzo[def]chrysene-8-one, 4-formylchrysene-5-carboxylic acid	[28]
*Hydrogenophaga* sp. PYR1	Anaerobic	Benzo[a]pyrene	5-Ethylchrysene, pyrene, 1H-Phenalen-1-one, phenanthrene, benzoic acid,2-hydroxy-phenyl ester, naphthalene, 1,2,3-trimethyl-4-propenyl	[43]
*Pseudomonas* sp. JP1	Anaerobic	Benzo[a]pyrene	1,12-Dimethyl-benz[a]anthracene/7,8,9,10-tetrahydrobenzo[a]pyrene/5-ethylchrysene, chrysene/benz[a]anthracene	[46]
*Klebsiella* sp. LZ6	Anaerobic	pyrene	Pyrene, 4,5-dihydro-, phenanthrene, allyl tert-butyldimethyl phthalate, 2′-hydroxypropiophenone, 4-hydroxycinnamate, p-Cresol, 4-hydroxybenzyl alcohol	[45]

Another limiting factor of microbial degradation is the bioavailability of PAH molecules. The hydrophobicity of the PAH compounds is related to the low aqueous solubility of the PAH pollutants presented in the soils, leading to the absorption of the PAHs to non-aqueous phase liquid (NAPL) of the polluted soils. This causes low bioavailability of the PAH compounds. The occurrence of PAH degradation by bacteria requires the solubilization of PAH pollutants to allow the bacterial metabolism of PAHs. There is a tendency of the partitioning of PAHs to the cell wall while transporting into the cell through passive transport. High bioavailability of the PAH pollutants enables more PAHs to cell transportation [51]. The factors affecting the solubility of PAH in water include temperature and molecular weight of PAHs. Elevated temperature can increase the solubility of PAHs but increased molecular weight of PAH pollutants can reduce their aqueous solubility. The reduction of the PAH bioavailability is also caused by the aging process where the sorption process becomes less reversible with longer PAH molecules [22]. The decrease in the metabolic rate of microorganisms under high salinity conditions is caused by the denaturation of proteins and enzymes of the microbes, reducing the microbial degradation rate of PAHs. The study by Minai demonstrates higher PAH degradation at 0% NaCl compared to 5% NaCl [52].

## Strategies for Biodegradation of PAH

There are two approaches in bioremediation of sites contaminated by PAH pollutants including in-situ bioremediation with lower disturbance and ex situ bioremediation. One of the ex situ bioremediation methods is using bioreactors. The site environment conditions are often not favorable for the microorganisms to perform degradation of PAH pollutants; hence, different methods have been introduced to improve the microbial degradation. The acceleration of microbial degradation of pollutants can be achieved through landfarming where oxygen, water, and nutrients are supplied through bulking, irrigation, and application of fertilizers respectively. Biostimulation is where the stimulation of indigenous microorganisms feeding on the pollutants occurs through supplementary of oxygen, nutrients, and fertilizers to increase the degradation rate by microorganisms. The added nutrients can facilitate the growth and activities of the microorganisms hence promoting the microbial degradation of PAHs. Exogenous microorganisms can also be introduced to the polluted sites to carry out the degradation process of PAH pollutants without considering the supply of nutrients, known as bioaugmentation, to enhance the microbial catabolic capacity [1, 22]. The advantages of using bacterial communities in degrading bacteria are the enhanced bacterial diversity in soils where the ability in metabolism and degradation of different PAHs differs among the bacteria. Apart from that, PAH degradation and removal can be enhanced through utilization of bacterial communities due to increases in the gene copies of ring-hydroxylating enzymes such as NidA and nahAC. The increases in PAH bioavailability are caused by increased aqueous solubility of PAHs by biosurfactant; hence, the addition of biosurfactant-producing bacteria can improve PAHs degradation [35]. Zeneli et al. proved that biostimulation and bioaugmentation-biostimulation increased the removal efficiency of PAHs from refinery solid waste up to 59% and 87%, respectively. The nutrients used for biostimulation in their study were monopotassium phosphate and ammonium nitrate, which most probably support microbial growth and activity, thus enhancing PAH degradation [53]. Also, Lang et al. reported that biostimulation along with bioaugmentation resulted in a pyrene degradation rate of 91.1% out of mixed PAHs; thereby reaffirming the potential of this integrated approach. Biostimulation alone and bioaugmentation alone resulted in 59.5% and 66.3% pyrene degradation, respectively. The PAH-degrading bacterium Rhodococcus erythropolis T902.1 was introduced in bioaugmentation treatment to further enhance the degradation mechanism. This present work indicates the promise that exists in the combination of biostimulation and bioaugmentation for increased PAH biodegradation efficiency in contaminated environments [54]. Both biostimulation and bioaugmentation are involved in the operation of bioreactors in degrading PAHs by the microbes. Optimum degradation of PAHs can be achieved by controlling the conditions within the bioreactors. Increased organic content of the soil under composting through adding agricultural waste and manure can increase the population of microbes present in the soil, facilitating the degradation of PAHs along with the degradation of agricultural waste added. Composting belongs to biostimulation, and it is revealed that composting methods is suitable for the HMW PAH degradation [6]. Rhizoremediation, which belongs to phytoremediation, involves remediation of PAH-contaminated soils by rhizosphere microorganisms. Nutrients such as organic acids, amino acids, and carbohydrates are supplied to the microorganisms by the plant roots, which promotes the growth of bacteria hence improving the PAH degradation by microbes. The PAH degradation by microbes can benefit the plants with decreasing phytotoxicity due to the removal of PAH contaminants [6, 55].

## Pros and Cons of Microbial Degradation

The main advantages of utilizing bacteria in degrading the PAH pollutants are the eco-friendliness and the low carbon footprint of the biodegradation methods, added that this method is also cost-efficient. The transformation of hazardous pollutants into non-hazardous products help remove the pollutants under microbial metabolism and degradation. The bioremediation of PAHs using microbes does not involve use of chemicals so further treatment of contaminated materials is not required. Another advantage of PAH bioremediation is it can be carried out at the contaminated site with minimal disturbance to the site environment [56, 57]. One of the limitations of microbial degradation of PAHs is the degradation process by bacteria can be easily affected by various abiotic factors like pH, temperature, and available oxygen, as these factors can affect the enzymic activity of the bacteria during PAH degradation. The contaminated site conditions often limit bacterial growth with the absence of oxygen and nutrients. The treatment period for the pollutants through bioremediation is also relatively slow compared to other remediation methods. The research on the implementation of the bioremediation of PAH pollutants at field scale is still required [56–58].

## Future Perspectives

One of the present difficulties in bioremediation of PAHs by microorganisms is the upscaling of microbial degradation from laboratory studies to field application, as most of the available studies on biodegradation of PAHs are conducted under controlled conditions in a laboratory [42]. To apply these results in practice, it is necessary to first take care of the environmental conditions, which include soil composition, moisture content, and temperature, as well as the concentration of pollutants. This demands large-scale field experiments to optimize the application of microbial bioremediation under actual environmental conditions. Moreover, PAH pollutants’ bioavailability is crucial for improving their microbial degradation. Chemotactic bacteria that can migrate toward the pollutants greatly enhance the bioavailability of PAHs in most contaminated environments. Thus, the study of bacterial chemotaxis mechanisms is a significant field of research that may help improve the efficiency of microbial degradation processes. Another potential area of future research is nanoparticle PAH biodegradation. Nanoparticles can improve the interaction between bacteria and pollutants, thereby accelerating degradation, by increasing the available surface area for adsorption. Such a novel approach may considerably uplift the bioremediation applications, especially in high persistence PAH compound environments where conventional means struggle to PAH twice as hard to degrade [59]. Mitigation of PAH pollution at the source is more than bioremediation. Emission reduction from petroleum refining, coal burning, and other sources of PAH compounds in the environment constitute basic strategies. Stringent regulations on industrial emissions, cleaner production technologies, and alternative energy sources would drastically reduce PAH compounds [60]. Other industrial housekeeping practices to be improved include the adoption of best practices in waste disposal and spill containment to prevent accidental releases from contaminating the environment with PAHs. Together with effective bioremediation techniques, these steps may considerably mitigate the adverse environmental and health effects of PAH pollutants and thus offer a much more sustainable approach to deal with PAHs contamination [61].

## Conclusion

Bioremediation of PAHs by microorganisms like bacteria is an effective remediation method of PAHs at the contaminated sites, added that this method is eco-friendly and cost-efficient. Bacterial strains capable of degrading PAHs are isolated from the PAH-contaminated sites, and their degradation ability on HMW PAHs and mixture of PAHs are being studied. Aerobic biodegradation of PAHs is common however in most of the cases the PAH-contaminated sites are always under anoxic conditions so anaerobic biodegradation of PAHs is also reviewed in this study. The utilization of microbial consortium in remediation and degradation of PAHs is promising in achieving high efficiency of PAH degradation with higher versatility of PAH metabolism by different bacteria in the consortium. There are several factors affecting the bacterial degradation of HMW PAHs including temperature, pH, and nutrients as these factors can influence the microbial activity and metabolism of bacteria, where the optimum conditions are normally neutral pH and 37 $^\circ{\rm C}$. Despite the recalcitrancy and low bioavailability of HMW PAHs, different approaches may be applied to enhance bacterial degradation by bioaugmentation and biostimulation. Under the method of bioaugmentation, addition of biosurfactant-producing bacteria can enhance dissolved HMW PAHs due to their poor aqueous solubility and low bioavailability. Future studies should include optimization of microbial consortia, genetic engineering approaches directed towards the reduction of degradation, and investigation of the combined application of bioaugmentation with other remediation technologies. Long-term studies in field conditions are also needed to evaluate the long-term efficiency and ecological impact of the strategies.

## Data Availability

Data is available upon request.
